# Nontargeted Metabolomics to Understand the Impact of Modified Atmospheric Packaging on Metabolite Profiles of Cooked Normal-pH and Atypical Dark-Cutting Beef

**DOI:** 10.3390/metabo14100532

**Published:** 2024-10-02

**Authors:** Keayla M. Harr, Noah Jewell, Gretchen G. Mafi, Morgan M. Pfeiffer, Ranjith Ramanathan

**Affiliations:** Department of Animal and Food Sciences, Oklahoma State University, Stillwater, OK 74078, USA; keayla.harr@okstate.edu (K.M.H.); noah.jewell@okstate.edu (N.J.); gretchen.mafi@okstate.edu (G.G.M.); morgan.pfeiffer@okstate.edu (M.M.P.)

**Keywords:** cooked color, beef, dark-cutting, metabolomics, packaging

## Abstract

**Background**: Limited knowledge is currently available on the effects of modified atmospheric packaging (MAP) on the metabolite profiles of cooked beef. The objective was to evaluate the impact of packaging on the cooked color and cooked metabolite profile of normal-pH (normal bright-red color) and atypical-dark-cutting beef (inherently slightly dark-colored) *longissimus lumborum* muscle. **Methods**: Normal-pH (pH 5.56) and atypical dark-cutting (pH 5.63) loins (*n* = 6) were procured from a commercial meat processor. Steaks were randomly assigned to one of three different packaging methods: vacuum packaging, carbon monoxide (CO-MAP), and high oxygen (HiOx-MAP). Following 5 d of retail display, steaks were cooked to 71 °C on a clamshell-style grill, and samples were collected for untargeted metabolites using gas-chromatography mass spectrometry. **Results**: Raw atypical dark-cutting steaks were less red (*p* < 0.05) than raw normal-pH steaks. However, there were no differences in internal cooked color between normal-pH and atypical dark-cutting steaks. Steaks packaged in HiOx-MAP steaks had a lower (*p* < 0.05) cooked redness than vacuum and CO-MAP steaks. A total of 129 metabolite features were identified in the study. Serine and tryptophan were over-abundant in cooked atypical dark-cutting beef compared to raw atypical samples. Citric acid levels were greater in HiOx-MAP packaged beef compared with VP both in normal and atypical dark-cutting beef after cooking, while no differentially abundant metabolites were shared between vacuum and CO-MAP steaks after cooking. **Discussion**: A slight increase in pH did not influence metabolite profiles in different packaging. However, there were packaging effects within normal and atypical dark-cutting beef. **Conclusions**: This study suggests that packaging conditions change metabolite profiles, which can influence cooked metabolites. Therefore, the metabolomics approach can be used to better understand cooked color defects such as premature browning.

## 1. Introduction

A bright cherry-red color is preferred by consumers when they purchase beef steaks and ground beef at retail. Therefore, any deviation from the consumer-accepted color of beef leads to decreased consumer purchasing [1]. Atypical dark-cutting is a color deviation from normal bright cherry red. Lower redness and darker color decrease consumer willingness to purchase beef at retail [2]. Atypical dark-cutting beef is characterized by a slightly elevated postmortem muscle pH (range from 5.8 to 6.0) compared to normal pH beef (range from 5.0 to 5.6). The cause of atypical dark-cutting beef is widely unknown. However, previous research has indicated a lower abundance of enzymes involved in glycolytic metabolism [3] and lower glycogen content [4], leading to a slightly elevated pH. In addition, atypical dark-cutting beef has greater oxygen consumption and metmyoglobin-reducing activity than normal-pH beef [3,5]. Furthermore, atypical dark-cutting beef has an upregulation of aconitic acid (involved in the tricarboxylic acid cycle) while having a downregulation of lactose and maltose compared with normal beef [3]. Proteomics and metabolomics studies noted that atypical dark-cutting beef had more changes in protein abundance (36 proteins) than metabolites (eight), suggesting the role of both protein and metabolic regulators leading to the atypical dark-cutting status [3]

Packaging meat for preservation and transport has been performed for centuries. The introduction of case-ready packaging has allowed beef purveyors to modify the gas composition within packages to increase shelf-life. Packaging atypical dark-cutting beef in high-oxygen modified atmospheric packaging (HiOx-MAP; 80% oxygen) and carbon monoxide (0.4% CO) MAP has been shown to improve the redness of traditional dark-cutting and atypical dark-cutting beef [5,6,7]. Previous studies also noted that biochemical properties of meat packaged in different atmospheric conditions, such as aerobic or anaerobic conditions, influence muscle oxygen consumption, metmyoglobin-reducing activity, lipid oxidation, and nicotinamide-adenine dinucleotide reduced form (NADH) content [6,8,9,10]. However, very little research has evaluated the impact of packaging on the biochemistry of cooked beef steaks.

In addition to the effects of packaging on biochemical properties, different gas compositions can promote cooked color issues such as premature browning [11,12]. More specifically, meat packaged in HiOx-MAP (80% oxygen and 20% carbon dioxide) predisposes myoglobin to more denaturation (well-done appearance) at temperatures below 71 °C [13,14]. Hence, consumers who use visual clues to determine the degree of doneness will be consuming undercooked meat. Currently, limited knowledge exists on the impact of cooking on metabolite profiles when packaging steaks in varying forms of MAP in conjunction with a slightly elevated meat pH. We hypothesize that the slightly elevated pH in conjunction with packaging will impact the metabolome of steaks following retail display and cooking. Therefore, the objective of this study was to evaluate the impacts of a slightly elevated pH and different packaging methods on the cooked color and cooked metabolite profile of beef *longissimus lumborum* steaks from normal and atypical dark-cutting beef after cooking to 71 °C.

## 2. Materials and Methods

### 2.1. Raw Materials and Packaging

Normal (mean pH = 5.56) and atypical dark-cutting (mean pH = 5.63; range 5.6 to 5.9) strip loins (*longissimus lumborum*; *n* = 6/loin type) were procured from a USDA-inspected beef processor, 24–36 h postmortem. Strip loins designated as atypical dark-cutting were selected based on having a visually dark-colored ribeye at the time of grading and a pH below 5.9. Loins were transported following fabrication to the Food and Agriculture Products Center at Oklahoma State University located in Stillwater, Oklahoma, repackaged in 3-mil high barrier vacuum package bags, and were wet aged for 14 d at 4 °C. Immediately following aging, the loins were sliced anterior to posterior with steaks being randomly designated for one of three different packaging types: vacuum package, HiOx-MAP (high-oxygen modified atmospheric packaging; 80% oxygen and 20% carbon dioxide), and CO-MAP (carbon monoxide modified atmospheric packaging; 0.4% carbon monoxide, 69.6% nitrogen, and 30% carbon dioxide). Additionally, a single steak was fabricated from each loin for the initial raw material analysis.

Following fabrication, steaks designated for vacuum packaging were immediately placed into 3-mil high barrier vacuum package bags (0.6 cm^3^ O_2_/645.16 cm^2^/24 h at 0 °C) and sealed with a vacuum packager (Multivac C5000; Multivac, Kansas City, MO, USA). After slicing, steaks designated for MAP packaging were placed into Rock-Tenn DuraFresh rigid trays (22.2 cm × 17.1 cm × 4.5 cm; RockTenn Company, Norcross, GA, USA) with a pre-placed soaker pad on the bottom of the tray. A semiautomatic Mondini tray sealing machine (Model VC/VG-5, G; Mondini, Cologne, Italy) was used to seal the trays with a Cryovac multilayer clear barrier film (LID 1050 film, 1 mil, <20 cm^3^ O_2_/m^2^/24 h; Cryovac Sealed Air, Duncan, NC, USA). Each tray was flushed with its respective gas blend using certified gas blends from Stillwater Steel and Supply (Stillwater, OK, USA). To ensure the proper gas composition had been achieved, the gas compositions were read on packages 1 h after packaging with a headspace analyzer (Mocon PAC Check Model 333; Mocon Inc., Minneapolis, MN, USA). Following packaging, all steaks were placed into coffin-style retail display cases at 3 ± 1.5 °C under continuous fluorescent lighting (Phillips fluorescent lamps, 12-watt, color temperature (3500 K; Phillips, Shanghai, China) for 5 d.

### 2.2. Cooking and Rest Period

Before cooking, steaks were allowed to equilibrate to room temperature for 30 min. After steaks were taken out of the packaging and the raw color was read, they were placed onto clamshell style griddles (Lean Mean Grilling Machine; George Foreman, Beachwood, OH, USA). The internal temperature was monitored throughout the cooking process using a thermometer (Super-Fast Thermapen; Thermoworks, American Fork, UT, USA) until an internal temperature of 71 °C (medium degree of doneness) was achieved. Following cooking, steaks were allowed to rest for 3 min on a wire rack prior to being cut for cooked color analysis.

### 2.3. Raw and Cooked Color

The raw surface color was measured on d 0 of the retail display (without packaging effect). On d 5 of the retail display, all steaks were taken out of the packages and immediately evaluated for objective color using a handheld HunterLab 4500L MiniScan EZ spectrophotometer (2.5-cm aperture, illuminant A, 10° standard observer angle, HunterLab Associates, Reston, VA, USA). The spectrophotometer was calibrated before use with the manufacturer-provided black and white tiles. Three reads were taken across the retail surface of each steak, and the three reads were averaged for each steak. The CIE L*, a*, and b* were recorded, along with the spectra from 400 to 700 nm. Chroma values were calculated based on a* and b* [15].

Previous studies have shown that carbon monoxide requires approximately 96 h (4 d) to fully saturate myoglobin [16]. Hence, steaks packaged and displayed for 5 d were used for cooked analysis. Internal cooked color was evaluated on steaks immediately after the 3-min rest period. Prior to reading the color, steaks were sliced horizontally through the center of the steak to give two halves. Two exposed surfaces to air from each half-piece were placed side by side to cover the measuring port of the HunterLab MiniScan spectrophotometer HunterLab Associates, Reston, VA, USA). Three color readings were recorded to characterize internal cooked surface color. Chroma values were calculated based on a* and b* values [13]. When taking readings with a HunterLab MiniScan, the machine automatically records the absorbance spectra from 400 to 700 nm, which can be used for further analysis. The ratio of 630 ÷ 580 nm was calculated to characterize the degree of doneness according to the American Meat Science (AMSA) Meat Color Guidelines [15]. A ratio closer to 1.0 indicates a more well-done, cooked color. Samples were also taken from raw and cooked samples for metabolomics analysis.

### 2.4. Metmyoglobin Reducing Activity

Sodium nitrite-mediated reducing activity was used to measure the metmyoglobin-reducing activity of the cooked steaks [17]. Steak halves were allowed to cool to room temperature following cooking. Steaks were sliced parallel to the external cooked surface to expose the interior portion of the steak. One-half piece with the internal cooked surface was submerged for 20 min in a 0.3% *w*/*v* sodium nitrite (Sigma Aldrich, St. Louis, MO, USA) solution at room temperature. After 20 min, samples were blotted dry with a paper towel to remove any excess solution. A HunterLab MiniScan EZ 4500L (2.5 cm aperture, illuminant A, 10° standard observer angle was used to measure the spectra from 400 to 700 nm by taking 3 reads across the internal cooked surface of the submerged samples. Metmyoglobin-reducing activity was calculated as the ratio of 630/580 nm. A larger ratio indicates greater metmyoglobin-reducing activity [15].

### 2.5. Nontargeted Metabolomics Analysis

Raw samples for metabolomics analyses were collected prior to packaging from each loin used on the initial day of the retail display to establish the base for the metabolome of the raw product. On d 5, following cooking, samples were taken from the interior of the steak to characterize the cooked metabolome. For both raw and cooked samples, approximately 0.5 g of sample was collected, packed into microcentrifuge tubes, and stored at −80 °C until analysis at the National Institute of Health West Coat Metabolomics Center at the University of California, Davis. Previously published methodology was used to extract metabolites [18]. Briefly, 1 mL of a degassed 3:3:3 *v*/*v*/*v* solution of acetonitrile/isopropanol/water mixture was added to 10 mg of the sample. After 30 s of homogenization of the sample mixture, samples were shaken at 4 °C for 6 min and centrifuged for 2 min at 14,000× *g*. Furthermore, an internal standard of methyl esters (2 µL of 1 mg/mL) was added to the mixture. Samples were then dried using nitrogen gas and derivatized for trimethylsilylation of acidic protons using methyloxolane in pyridine and N-methyl-N (trimethylsilyl) trifluoroacetamide. Metabolites were separated using a gas chromatography-mass spectrometer. All other details about metabolomics profiling are included in previously published research [18].

### 2.6. Statistical Analysis

For all raw and cooked color and metmyoglobin-reducing activity analyses, data were averaged for each sample and were analyzed as a split-plot design. The loin type served as the whole plot, while the steak within the packaging type served as the subplot. The Glimmix procedure of SAS (version 9.4; SAS Institute, Cary, NC, USA) was utilized to generate least square means using the Kenward Roger adjustment for denominator degrees of freedom. The level of significance was set at an α of 0.05 for all color analyses.

Metabolites were evaluated using Metaboanalyst 6.0. All metabolite peak intensities were normalized by median, log-transformed, and scaled with auto-scaling prior to being analyzed. Pairwise comparisons were conducted between the raw and cooked samples of the three different packaging types using *t*-tests based on the raw *p*-value. Comparisons within packaging type and packaging/loin type combinations utilized Fisher’s LSD to determine significantly different abundant metabolites between treatments. A *p* < 0.05 was considered significant for all metabolite analyses. Overall differences in metabolites are expressed in supervised projections to latent structure-discriminant analysis (PLS-DA) plots to demonstrate the separation of metabolites between treatments. Additionally, pairwise comparisons between the cooked and raw treatments for each loin type were used to generate Venn diagrams showing the significantly different metabolites shared between the groups.

## 3. Results

### 3.1. Raw Color Attributes

There were no significant interactions between loin type and packaging type for raw color attributes; therefore, the main effects are reported (Table 1). As expected, atypical dark-cutting steaks had a lower (*p* < 0.05) L* value and were darker in color prior to cooking than normal steaks. There were no (*p* > 0.05) differences in raw L* values for packaging type prior to cooking. HiOx-MAP steaks had a higher (*p* < 0.05) a* (redness) and chroma value than CO-MAP and vacuum packaged steaks prior to cooking (HiOx-MAP > CO-MAP > vacuum; *p* < 0.05). However, there were no (*p* > 0.05) differences in loin type for both a* and chroma for the raw steaks.

### 3.2. Cooked Color Attributes

Cooked internal color analyses noted no significant interactions between loin type and packaging for L*, a*, chroma, and the ratio of 630 and 580 nm (Table 1). There were no (*p* > 0.05) differences for loin type for all the color parameters evaluated. However, there was a packaging main effect significance for the cooked internal color redness. CO-MAP and vacuum pack-aged steaks had higher (*p* < 0.05) a* and chroma values than HiOx-MAP steaks. Furthermore, HiOx-MAP steaks had a lower (*p* < 0.05) ratio of 630/580 nm and exhibited a more well-done internal cooked color than CO-MAP and vacuum-packaged steaks. There was no (*p* > 0.05) difference in cooked internal L* for packaging type.

There were no (*p* > 0.05) differences in metmyoglobin-reducing activity for the comparison of cooked normal-pH and atypical dark-cutting steaks (Table 1). However, there was a significant packaging type that had a main effect on metmyoglobin-reducing activity. Vacuum-packaged steaks had a higher (*p* < 0.05) metmyoglobin-reducing activity than HiOx-MAP steaks. However, CO-MAP packaged steaks were similar (*p* > 0.05) to vacuum-packaged steaks.

### 3.3. Effects of Muscle Type on Metabolite Profile

**Figure 1:**
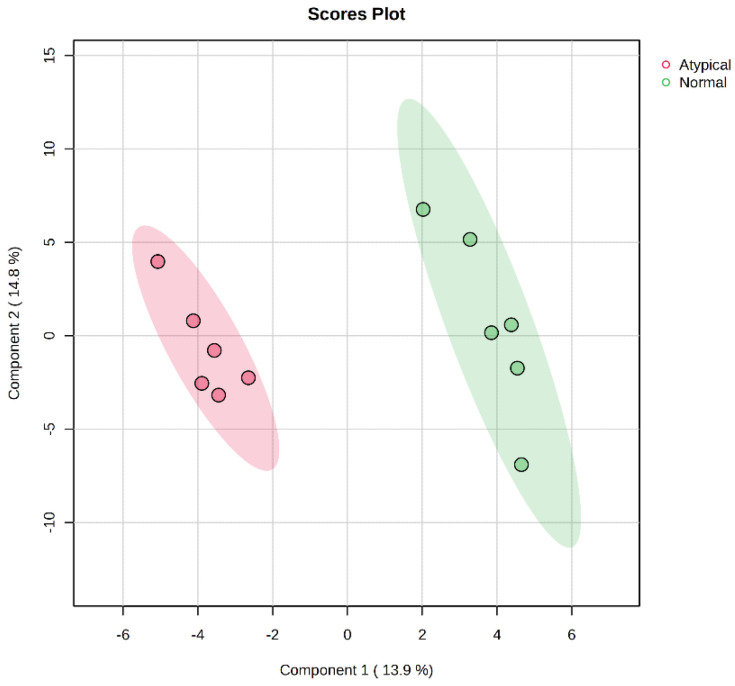
Projections to latent-discriminant analysis plot (PLS-DA) of the metabolites present in the raw normal and raw atypical dark-cutting beef *longissimus lumborum* at the beginning of retail display. Red color denotes atypical dark-cutting samples, while green denotes normal samples.

Untargeted metabolomics analysis identified 367 features, with 129 metabolites identified within the metabolite library. The plot of PLS-DA scores showed a separation between the metabolite profiles of raw normal and raw atypical dark-cutting samples (Figure 1 and Table 2). There were nine differentially abundant metabolites present in the comparison of raw normal to raw atypical dark-cutting beef. Of those, three metabolites were less abundant in atypical dark-cutting beef than in normal beef, including lactic acid and palmitoleic acid. Arachidonic acid, dehydroascorbic acid, heptadecanoic acid, linoleic acid, phenylethylamine, threonic acid, and 2-hydroxyglutaric acid were over-abundant in atypical dark-cutting beef compared to normal beef. Of the significantly different metabolites, four metabolites are involved in fatty acid metabolism, while two metabolites were involved in the TCA cycle and glycolysis.

### 3.4. Effects of Muscle Type and Packaging on Cooked Metabolite Profiles

**Figure 2:**
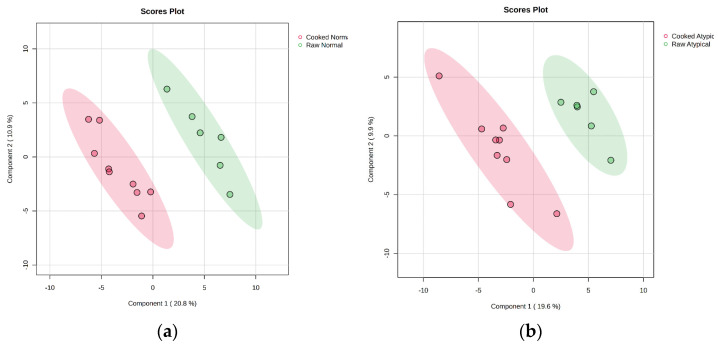
Projections to latent-discriminant analysis plot (PLS-DA) of the metabolites present in (**a**) raw normal *longissimus lumborum* at the beginning of retail display and normal *longissimus lumborum* following 5 d of retail display and cooking to 71 °C and (**b**) raw atypical dark-cutting *longissimus lumborum* at the beginning of retail display and atypical dark-cutting *longissimus lumborum* following 5 d of retail display and cooking to 71 °C. Red color in both figures corresponds to the respective cooked product, while green indicates raw.

The current study showed no differences between cooked color attributes of nor-mal-pH and atypical dark-cutting beef. Hence, data for each muscle type was analyzed separately for packaging. PLS-DA plot evaluation indicates distinct differences in the cooked normal and cooked atypical samples (Figure 2) when packaging type is not included. PLS-DA plot analysis also indicates a separation between the three different types of packaging when cooked atypical dark-cutting and normal samples are independently evaluated.

A comparison of raw atypical dark-cutting beef to cooked atypical dark-cutting beef demonstrated 14 significantly different metabolites (Table 3). Aspartic acid, isoleucine, leucine, phenylalanine, serine, and tryptophan were over-abundant in the cooked atypical dark-cutting beef compared to raw atypical dark-cutting beef. In addition, carbohydrate metabolites such as cellobiose, isomaltose, and lyxose were over-abundant in cooked atypical dark-cutting beef. Only glycyl-proline was over-abundant in the cooked atypical dark-cutting beef. Dehydroascorbic acid, nicotinamide, squalene, and threonic acid were all less abundant in atypical dark-cutting beef that was cooked compared to raw atypical dark-cutting beef.

Comparing raw normal beef to normal beef following 5 d of retail display and cooking at 71 °C yielded 23 significantly different metabolites (Table 4). Amino acids that were identified as being overabundant in normal beef that was cooked included aspartic acid, cysteine, glutamic acid, isoleucine, leucine, methionine, phenylalanine, threonine, tryptophan, tyrosine, and valine. Xylulose was overabundant in cooked normal beef, while lactic acid was less abundant in cooked normal beef. Glycerol was also overabundant in cooked normal beef. Guanosine, dehydroascorbic acid, and creatine were less abundant in cooked normal beef compared to raw normal beef.

### 3.5. Packaging Comparison of Atypical Dark-Cutting Beef

**Figure 3:**
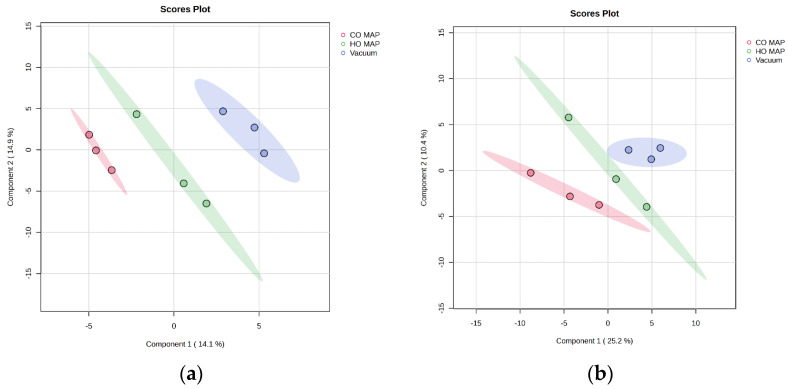
Projections to latent-discriminant analysis plot (PLS-DA) of the metabolites present in cooked (**a**) atypical dark-cutting beef and (**b**) normal in different packaging. The red color in both plots corresponds to CO-MAP packaged steaks, green corresponds to HO-MAP (high-oxygen MAP), and blue denotes vacuum packaged steaks.

Pairwise comparisons between packaging types for atypical and normal beef following cooking were conducted separately, as there was no muscle-type interaction (Table 5 and Figure 3a). Comparing atypical dark-cutting beef packaged in CO-MAP to atypical dark-cutting beef packaged in HiOx-MAP indicated 7 differentially abundant metabolites. Of those metabolites, threonic acid and trans-4-hydroxyproline were over-abundant in atypical dark-cutting beef packaged in CO-MAP, while the remaining metabolites, including citric acid, fumaric acid, maltose, maltotriose, and xanthine, were less abundant in CO-MAP than HiOx-MAP atypical dark-cutting steaks. Similarly, citric acid, maltose, maltotriose, and xanthine were over-abundant in atypical dark-cutting HiOx-MAP compared to vacuum-packaged atypical dark-cutting steaks. Threonic acid was the only metabolite with less abundance in HiOx-MAP atypical dark-cutting steaks than in atypical dark-cutting steaks packaged in a vacuum. There were no significantly different metabolites between atypical dark-cutting beef packaged in CO-MAP and atypical dark-cutting beef packaged in a vacuum.

### 3.6. Packaging Comparison of Cooked Normal-pH Beef

Four metabolites were significantly different in cooked normal-pH steaks in three different forms of packaging (Table 6 and Figure 3b). Citric acid and fumaric acid were less abundant in normal steaks packaged in CO-MAP than in normal steaks packaged in HiOx-MAP. However, citric acid and fumaric acid were over-abundant in normal steaks in HiOx-MAP compared to normal steaks packaged in a vacuum. Similarly, malic acid and threonic acid were more abundant in normal steaks packaged in CO-MAP than normal steaks packaged in HiOx-MAP and were less abundant in normal high-oxygen MAP steaks compared to normal vacuum packaged steaks. There were no significantly different metabolites when comparing normal steaks in CO-MAP and normal steaks in vacuum packaging.

## 4. Discussion

Limited research has evaluated atypical dark-cutting beef to understand the underlying muscle metabolism and its impacts on muscle color. Previous research using beef at muscle pH above 6.4 (typical dark-cutting pH range) indicated a lower abundance of glycolytic enzymes and metabolites compared to normal pH [19,20,21,22]. However, in atypical dark-cutting beef with only a slightly elevated pH, there were more changes in protein expression than metabolites related to glycolysis and tricarboxylic pathways [3]. Atypical dark-cutting beef has lower glycogen content than normal pH. In support, atypical dark-cutting beef had lower lactic acid than normal pH. Hence, other energy sources, such as amino acids and fatty acids, might have been used for maintaining energy homeostasis. In the current study, several fatty acid metabolites, such as arachidonic acid, heptadecanoic acid, and linoleic acid, were over-abundant in the atypical dark-cutting beef compared with normal-pH beef. A previous study in dark-cutting beef showed greater levels of 4-aminobutyric acid (neurotransmitter) than normal-pH beef [23]. In the current study, phenylethylamine (neurotransmitter simulant) was upregulated in atypical dark-cutting beef at a higher pH than normal. In addition, there was an overabundance of 2-hydroxyglutaric acid, a tricarboxylic acid cycle metabolite, further suggesting that atypical dark-cutting beef is due in part to alterations in energy metabolism and a shift from glycolysis.

### 4.1. Impact of Packaging Type and Cooking on Metabolites

Cooking can accelerate biochemical reactions such as oxidation, denaturation of proteins, condensation of amino acids, and release of free amino acids. A recent study noted that cooking influences protein profiles differentially in HiOx-MAP and vacuum packaging [11]. Protein and metabolite profiles are interrelated; hence, metabolite profiles can also be different in different kinds of packaging. However, limited studies have determined the impact of packaging and cooking on metabolite profiles. 

Beef cooked to an internal temperature of 71 °C will have a reddish-pink-colored interior, which consumers will use to determine a medium degree of doneness. In the current study, both atypical dark-cutting and normal steaks packaged in HiOx-MAP exhibited premature browning characteristics (grayish-brown interior) and were less red internally than vacuum and CO-MAP steaks. Previous research reported high incidences of premature browning in both ground beef and beef steaks packaged in HiOx-MAP, primarily attributed to the high levels of oxygen that are bound to the myoglobin, causing myoglobin to denature at lower temperatures [13,17,24,25]. In addition, HiOx-MAP packaging allows oxygen to diffuse into the interior of steaks, further perpetuating the problem of premature browning. We hypothesized that the slight increase in pH, coupled with the dark color of the atypical dark-cutting, could potentially decrease the impact of premature browning in the HiOx-MAP packaging. Previous studies have noted traditional dark-cutting beef to have a redder interior when cooked [26,27,28,29], suggesting that the darker color in atypical steaks might not denature as fast as normal steaks. Steaks packaged in HiOx-MAP had less metmyoglobin-reducing activity than vacuum-packaged steaks, making them less color-stable in both the raw and cooked state.

Seven metabolites were differently present in atypical dark-cutting in different packaging. However, four metabolites were differently present in normal pH steaks in various packages. Both CO-MAP and vacuum packages create anaerobic conditions. Interestingly, in atypical and normal-pH-cooked steaks, there were no differentially abundant metabolites. Previous research also noted that vacuum and CO-MAP resulted in similar oxygen consumption and metmyoglobin-reducing activity [5,30]. However, both muscle types had different profiles and abundance when comparing CO-MAP vs. HiOx-MAP and vacuum vs. HiOx-MAP. In contrast to proteins, metabolites are more stable when heated. Further, some of the chemical reactions can be accelerated when the temperature of meat rises from 4 to 71 °C. In support, metmyoglobin-reducing activity still occurred after cooking [17,26,31].

Citric acid is a part of the tricarboxylic acid (TCA) cycle and has previously been found to be upregulated in traditional, high-pH dark-cutting beef [20]. However, previous studies with atypical dark-cutting beef did not find citric acid to be impacted when compared to normal beef without the impact of packaging [3,32]. In the present study, steaks packaged in HiOx-MAP had a greater abundance of citric acid in both atypical dark-cutting and normal steaks post-cooking. This suggests that the added oxygen in HiOx-MAP could potentially play a role in citric acid metabolism. In addition to citric acid, fumaric acid was also found to be differentially abundant and upregulated in HiOx-MAP packaging, further suggesting the role of additional oxygen concentrations in citric acid formation. Alternatively, a greater amount of lipid oxidation can occur in HiOx-MAP packaging [7,33], which might lead to autoxidation, increasing the number of organic acids present.

A greater number of differentially abundant in raw and cooked normal-pH beef than in atypical raw and cooked beef suggests that greater glycogen levels, in turn, can influence cooked metabolites. Glycolytic metabolites were more abundant in normal-pH beef than in dark-cutting conditions. As expected, several amino acids were observed both in cooked normal and atypical dark-cutting beef. Currently, very few published studies are available on the roles of metabolites present in cooked steak on color properties. It is well known that enzymes retain some activity at cooking temperatures. Future research at different cooking temperatures, along with measuring activities of enzymes, will provide a better understanding of the role of metabolites in cooked color development. The current study suggests metabolomics can be used to understand cooked color defects like premature browning or persistent pinking.

### 4.2. Impact of Packaging and Metabolites on Other Meat Quality Attributes

While sensory analysis was not conducted in the present study, we can also make inferences about the amino acids found contributing to beef flavor and aroma. Free amino acids contribute to basic tastes associated with meat flavor, while heating peptides and 5’ ribonucleotides contribute to sour, bitter, and umami [34,35,36]. Aspartic acid, overabundant in both atypical and normal-pH samples that were cooked, is associated with umami flavor [34,37]. Isoleucine, leucine, and phenylalanine were overabundant in cooked normal and atypical dark-cutting beef and are associated with bitter flavor [34,38]. Cysteine and methionine, associated with meat-like sweet flavor [34,39,40], were over-abundant in normal beef but were not differentially abundant in atypical dark-cutting beef. In addition, threonine was also overabundant in cooked normal-pH beef and associated with sweet flavor and odor [34,41,42]. In the present study, no amino acids were differentially abundant in the pairwise comparisons of packaging types. However, previous research indicated that less isoleucine and methionine are present in strip steaks from normal-pH beef in HiOx-MAP than in both CO-MAP and vacuum steaks. Further, more sour and less umami flavors were reported when the same steaks were in HiOx-MAP [43]. The same study also noted a similar amount of phenylalanine, leucine, tyrosine, and tryptophan in CO-MAP and HiOx-MAP steaks but less in vacuum-packaged steaks. However, trained panelists found a greater amount of beefy flavor in vacuum-packaged steaks than in steaks packaged in HiOx- and CO-MAP. While it is unknown in the current study how the various metabolites contribute to the flavor and sensory experience, we can hypothesize that atypical dark-cutting and normal beef in different packaging types might result in differences in flavor profiles based on metabolite abundance.

## 5. Conclusions

Raw atypical dark-cutting beef has an up-regulation of metabolites related to fatty acids compared to raw normal-pH beef. However, normal and atypical conditions did not influence cooked color characteristics when packaged in vacuum, CO-MAP, and HiOx-MAP. Despite a slight increase in pH, atypical dark-cutting steaks had a lower internal cooked color indicative of premature browning. Raw and cooked normal-pH beef had more differentially abundant metabolites than raw and cooked atypical dark-cutting beef. There was a greater abundance of citric acid in cooked normal and atypical dark-cutting steaks packaged in HiOx-MAP. However, no differentially abundant metabolites were shared between CO-MAP and vacuum packages. The current study suggests that packaging conditions and glycogen content in beef before cooking influence the metabolite profiles of cooked steaks. In addition, metabolite characterization helps to understand cooked color defects like premature browning or persistent pinking.

## Figures and Tables

**Table 1:** Least square means for the raw and internal cooked color ^1^ and cooked metmyoglobin reducing activity ^2^ of atypical dark-cutting or normal beef *longissimus lumborum* steaks (*n* = 6/loin type) packaged in three different retail packaging.

	Raw Color			Cooked Internal Color			
	L*	a*	Chroma	L*	a*	Chroma	630 nm/580 nm
Loin type							
Atypical dark-cutting	42.76 ^b^	21.10	24.57	52.40	18.78	27.34	2.33
Normal	45.85 ^a^	21.79	25.92	50.28	18.59	28.00	2.34
Standard error:	0.90	0.70	0.79	0.79	0.49	0.50	0.09
*p*-value:	0.03	0.50	0.23	0.09	0.79	0.36	0.95
Packaging type							
CO-MAP	42.92	23.34 ^b^	26.87 ^b^	50.81	22.82 ^a^	31.10 ^a^	2.94 ^a^
HiOx-MAP	44.90	25.89 ^a^	31.21 ^a^	52.03	11.50 ^b^	21.33 ^b^	1.32 ^b^
Vacuum package	45.10	15.11 ^c^	17.65 ^c^	51.19	21.74 ^a^	30.59 ^a^	2.73 ^a^
Standard error:	0.95	0.82	0.97	0.70	0.56	0.61	0.11
*p*-value:	0.17	<0.01	<0.01	0.25	<0.01	<0.01	<0.01

^abc^ Least square means within a main effect and parameter lacking a common superscript differ (*p* < 0.05). ^1^ Raw and internal color measured using a HunterLab MiniScan EZ 4500L spectrophotometer. L* indicates lightness (a greater value indicates lighter color, while a lower value means darker color; a* value indicates redness (a greater value indicates more red while a lower value means less red; chroma calculated according to AMSA color guidelines and indicates red intensity where a lower value indicate less intense red color, while greater value means intense red color. The ratio of 630 over 580 nm indicates redness. A greater value indicates a red color, while a lower value indicates less red color. ^2^ Cooked metmyoglobin-reducing activity was conducted on samples following cooking and immersing in a 0.3% sodium nitrite solution for 20 min. A greater 630 nm/580 nm indicates greater metmyoglobin-reducing activity.

**Table 2:** Differentially abundant metabolites in raw atypical dark-cutting beef compared to raw normal beef at the beginning of retail display.

Metabolite	*p*-Value	Abundance (Atypical/Normal)	Role
2-hydroxygluataric acid	0.040	over	Lysine and tryptophan degradation
Arachidonic acid	<0.001	over	Fatty acid metabolism
Linoleic acid	0.003	over	Fatty acid metabolism
Heptadecanoic acid	0.026	over	Fatty acid metabolism
Threonic acid	0.025	over	Sugar acid
Phenylethylamine	0.002	over	Neurotransmitter
Dehydroascorbic acid	0.026	over	Antioxidant metabolism
Palmitoleic acid	0.046	less	Fatty acid metabolism
Lactic acid	0.014	less	Glycolysis

**Table 3:** Differentially abundant metabolites in cooked ^1^ and raw ^2^ atypical dark-cutting beef.

Metabolite	*p*-Value	Abundance (Cooked/Raw)	Type of Metabolite
Aspartic acid	0.019	over	Amino acid
Tryptophan	0.018	over	Amino acid
Glycyl-proline	0.005	over	Dipeptide
Isoleucine	0.032	over	Amino acid
Leucine	0.030	over	Amino acid
Serine	0.029	over	Amino acid
Phenylalanine	0.031	over	Amino acid
Isomaltose	0.010	over	Carbohydrate
Lyxose	0.003	over	Carbohydrate
Cellobiose	0.012	over	Carbohydrate
Squalene	0.005	less	Lipid
Dehydroascorbic acid	0.013	less	Vitamin (oxidized)
Nicotinamide	0.005	less	Vitamin
Threonic acid	0.033	less	Sugar acid

^1^ Cooked atypical dark-cutting beef following 5 d of retail display and cooking to 71 °C. ^2^ Raw atypical dark-cutting beef (pH slightly greater and darker in color) at the beginning of retail display.

**Table 4:** Differentially abundant metabolites in raw ^1^ and cooked ^2^ normal-pH beef.

Metabolite	*p*-Value	Abundance (Cooked/Raw)	Type of Molecule
Aspartic acid	0.028	over	Amino acid
Cysteine	0.005	over	Amino acid
Glutamic acid	0.007	over	Amino acid
Isoleucine	0.001	over	Amino acid
Leucine	0.001	over	Amino acid
Methionine	0.014	over	Amino acid
Phenylalanine	0.018	over	Amino acid
Threonine	0.007	over	Amino acid
Tryptophan	0.047	over	Amino acid
Tyrosine	0.031	over	Amino acid
Valine	0.002	over	Amino acid
3,6-anhydro-D-galactose	0.008	over	Carbohydrate
Lyxose	<0.001	over	Carbohydrate
N-acetylneuraminic acid	0.010	over	Carbohydrate
Xylulose	0.019	over	Carbohydrate
Glycerol	0.019	over	Lipid base
Glycerol-3-galactoside	0.037	over	Glycoside
Glycyl-proline	<0.001	over	Dipeptide
Hypoxanthine	0.046	over	Purine base
Creatinine	0.011	less	Metabolite
Dehydroascorbic acid	0.009	less	Vitamin (oxidized)
Guanosine	0.047	less	Nucleoside
Lactic acid	0.013	less	Organic acid

^1^ Cooked normal-pH beef following 5 d of retail display and cooking to 71 °C. ^2^ Raw normal-pH beef (normal bright red and pH 5.6) at the beginning of retail display.

**Table 5:** Effects of packaging on metabolites of cooked atypical dark-cutting beef.

Metabolite	CO vs. HO		CO vs. VP	HO vs. VP	
	*p*-Value	Abundance (CO/HO)	*p*-Value	*p*-Value	Abundance (HO/VP)
Citric acid	0.005	less	NS	0.002	over
Fumaric acid	0.002	less	NS	NS	-
Maltose	0.017	less	NS	0.009	over
Maltotriose	0.007	less	NS	0.044	over
Threonic acid	0.017	over	NS	0.017	less
Trans-4-hydroxyproline	0.031	over	NS	NS	-
Xanthine	0.003	less	NS	0.002	over

Pairwise comparisons were conducted using student *t*-tests in MetaboAnalyst 6.0. Packaging comparisons are solely for atypical dark-cutting beef *longissimus lumborum* steaks cooked to 71 °C from atypical dark-cutting beef strip loins following 5 d of retail display. NS indicates not significant (*p* > 0.05) for the comparison. Packaging abbreviations: CO = 0.4% carbon monoxide; HO = high-oxygen MAP; VP = vacuum packaging.

**Table 6:** Effects of packaging on metabolites of cooked normal-pH beef.

Metabolite	CO vs. HO		CO vs. VP	HO vs. VP	
	*p*-Value	Abundance (CO/HO)	Abundance	*p*-Value	Abundance (HO/VP)
citric acid	<0.001	less	NS	<0.001	over
fumaric acid	0.010	less	NS	<0.001	over
malic acid	0.002	over	NS	0.002	less
threonic acid	0.003	over	NS	<0.001	less

Pairwise comparisons were conducted using student *t*-tests in MetaboAnalyst 6.0. Packaging comparisons are for normal-pH beef *longissimus lumborum* steaks cooked to 71 °C from normal-pH beef strip loins following 5 d of retail display. No significant differences between CO and VP. NS indicates not significant (*p* > 0.05) for the comparison. Packaging abbreviations: CO = 0.4% carbon monoxide; HO = high-oxygen MAP; VP = vacuum packaging.

## Data Availability

Data will be available based on the request.
